# SARS-CoV-2 in Environmental Samples of Quarantined Households

**DOI:** 10.3390/v14051075

**Published:** 2022-05-17

**Authors:** Manuel Döhla, Bianca Schulte, Gero Wilbring, Beate Mareike Kümmerer, Christin Döhla, Esther Sib, Enrico Richter, Patrick Frank Ottensmeyer, Alexandra Haag, Steffen Engelhart, Anna Maria Eis-Hübinger, Martin Exner, Nico Tom Mutters, Ricarda Maria Schmithausen, Hendrik Streeck

**Affiliations:** 1 Institute for Hygiene and Public Health, Medical Faculty, University of Bonn, Venusberg-Campus 1, 53127 Bonn, Germany; manueldoehla@bundeswehr.org (M.D.); gero.wilbring@ukbonn.de (G.W.); christin.doehla@gmail.com (C.D.); esther.sib@ukbonn.de (E.S.); alexandra.haag@ukbonn.de (A.H.); steffen.engelhart@ukbonn.de (S.E.); martin.exner@ukbonn.de (M.E.); nico.mutters@ukbonn.de (N.T.M.); ricarda.schmithausen@ukbonn.de (R.M.S.); 2 Department of Microbiology and Hospital Hygiene, Bundeswehr Central Hospital Koblenz, Rübenacher Straße 170, 56072 Koblenz, Germany; 3 Institute of Virology, Medical Faculty, University of Bonn, Venusberg-Campus 1, 53127 Bonn, Germany; bianca.schulte@ukbonn.de (B.S.); beate.kuemmerer@uni-bonn.de (B.M.K.); enrico.richter@ukbonn.de (E.R.); patrick.ottensmeyer@ukbonn.de (P.F.O.); anna-maria.eis-huebinger@ukbonn.de (A.M.E.-H.)

**Keywords:** SARS-CoV-2, COVID-19, smear infection, environment, quarantine, airborne transmission

## Abstract

The role of environmental transmission of SARS-CoV-2 remains unclear. Thus, the aim of this study was to investigate whether viral contamination of air, wastewater, and surfaces in quarantined households result in a higher risk for exposed persons. For this study, a source population of 21 households under quarantine conditions with at least one person who tested positive for SARS-CoV-2 RNA were randomly selected from a community in North Rhine-Westphalia in March 2020. All individuals living in these households participated in this study and provided throat swabs for analysis. Air and wastewater samples and surface swabs were obtained from each household and analysed using qRT-PCR. Positive swabs were further cultured to analyse for viral infectivity. Out of all the 43 tested adults, 26 (60.47%) tested positive using qRT-PCR. All 15 air samples were qRT-PCR-negative. In total, 10 out of 66 wastewater samples were positive for SARS-CoV-2 (15.15%) and 4 out of 119 surface samples (3.36%). No statistically significant correlation between qRT-PCR-positive environmental samples and the extent of the spread of infection between household members was observed. No infectious virus could be propagated under cell culture conditions. Taken together, our study demonstrates a low likelihood of transmission via surfaces. However, to definitively assess the importance of hygienic behavioural measures in the reduction of SARS-CoV-2 transmission, larger studies should be designed to determine the proportionate contribution of smear vs. droplet transmission.

## 1. Introduction

The COVID-19 pandemic is one of the most important global public health threats since the Spanish Flu more than 100 years ago. Over 422 million cases and more than 5.8 million deaths have been reported so far [1]. The pandemic is a major challenge for environmental hygiene in both hospitals and homes. Special isolation and infectious disease wards have been established in hospitals and healthcare facilities, and whole households have been quarantined. Hence, comprehensive monitoring of the environment of healthcare facilities and households during pandemic outbreaks is vital to ensure both patient safety and public health [2]. 

COVID-19 is a disease of the upper airways [3]. It has been shown for SARS-CoV-2 that droplets (particles > 5 µm) can deposit on mucous surfaces of the upper respiratory tract and can be spread when coughing, sneezing, or speaking [2,4,5,6]. As a result, the main airborne transmission pathways of infectious SARS-CoV-2 are aerosols (particles < 5 µm) or droplets (particles > 5 µm) [7]. This is particularly important regarding indoor environments because small particles with a higher viral load may be carried over distances up to 10 m from the emission source and may even accumulate [8,9]. Viable SARS-CoV-2 particles could be found in the air of spaces that were used by SARS-CoV-2 infected individuals [10]. Additionally, SARS-CoV-2 RNA was found in the filters of the air filtration systems of COVID-19 wards [11]. Enhancement of ventilation in indoor environments is one way to reduce the risk of infection [12]. Nevertheless, in-field outbreak studies on the environmental transmission dynamics of SARS-CoV-2 show potential effects of fomite transmission. One of those studies was performed by Xu and co-workers on a cruise ship with 3711 passengers [13]. They were able to show that long-range airborne transmission routes and even central air conditioning systems are not necessarily the main drivers of a COVID-19 outbreak in a confined space. It can be assumed that close contact and fomites contribute to transmission effects [14,15]. Indeed, field studies of SARS-CoV-2 detected RNA on door handles and surfaces in a hospital or confirmed COVID-19 in other samples from the patient’s environment [2,16,17]. The RNA of the virus could also be detected on surfaces that are often touched by individuals in public spaces [18,19]. However, the prevalence and potential environmental transmission risks of SARS-CoV-2 in households where infected persons live with their families have not yet been sufficiently explored. 

One important underlying question is whether and how long virus particles can survive on various surfaces to enable human-to-surface-to-human transmission. To date, few cases of suspected transmission of SARS-CoV-2 from human to human via food, drinking water, or fomites have been reported [20,21,22]. 

Additionally, studies have shown that the viable virus remains detectable for hours or even days in inanimate surroundings like the air, on stainless steel and plastic surfaces [23,24], as well as in the urine and faeces of formerly positive patients [25,26,27]. SARS-CoV-2 RNA was detected in the stool of one of the first patients in the USA [25]. Sawaarn and Hait found a detectable viral load in faeces and wastewater in 39% of infected persons with diarrhoea and in 9% of individuals without diarrhoea [28]. This might be in line with the observation of Wang and colleagues, as well as Cheng and coworkers, describing that at least 2–10% of patients with COVID-19 show gastrointestinal symptoms such as diarrhoea and vomiting [29,30]. This is supported by surveillance data from the UK, in which only in February 2021, more than 10% of infected persons reported diarrhoea as a symptom. However, the reduced incidence of diarrhoea seems to be related to the emergence of new virus variants [31]. Since the present study was conducted in the early phase of the pandemic, it can be assumed that the wild-type virus strain was present. In contrast, Schmithausen and coworkers described persistent diarrhoea in 32% of tested persons [32]. A meta-analysis focusing on gastrointestinal symptoms revealed that this type of symptom occurs in 3-39% of COVID-19 cases [33]. A study by Greco and colleagues looked at the distribution of symptoms depending on age, sex, and disease outcome, among other factors. In this study, gastrointestinal symptoms occurred in 12.5% of infected individuals [34].

Following the wastewater pathway, SARS-CoV-2 RNA has already been found in wastewater [26], which has been demonstrated to be a reliable way of monitoring COVID-19 infections and variant spread [35,36]. Moreover, when SARS-CoV-2 infected persons with gastrointestinal symptoms excrete urine and faeces, SARS-CoV-2 can be identified in the immediate surroundings, such as the sanitary facilities. While the elimination of SARS-CoV-2 in wastewater is feasible by wastewater treatment [25,37], the possibility of faecal–oral recirculation of SARS-CoV-2 from siphons of washbasins, showers, and toilets to humans via droplets, aerosols, or even smear-infection is still unclear. To test this assumption, during our study we also collected in-field samples of siphons and toilets in private households of COVID-19 infected people. 

Exposure to and transmission of the virus primarily occurs at home [38]. and in cases of mild COVID-19 progression, home care is implemented to avoid hospital overload [39]. Consequently, contact persons of people who tested positive are also placed in pre-emptive home isolation before the onset of symptoms due to the risk of contagion [14,39]. Infected people and contact persons who live together as a family or in cohabitation thus find themselves in domestic quarantine with each other. Even with separate bathrooms and bedrooms, it is impossible to effectively and permanently distance oneself and maintain adequate hand hygiene.

The main aim of this study was to answer the question of how high the prevalence of contamination of air, wastewater, and specific surfaces was in households where at least one member is currently isolated due to a positive SARS-CoV-2 test result and to give useful recommendations for infection prevention. 

## 2. Material and Methods

### 2.1. Study Design Sample Size and Recruitment of Households

This study was conducted between 3 and 23 March 2020 in the German district of Heinsberg in North Rhine-Westphalia. We chose this district because at that time of the pandemic in Germany, its officially reported positive cases in this community were 3% (approx. 12,500 inhabitants at time of study period). In this community, carnival festivities around mid-February were followed by a massive outbreak of SARS-CoV-2. Actual research data indicate a level of 15.5% of individuals with a positive infection status in March 2020 [40]. In this district strict measures, including a voluntary curfew, were immediately taken to slow down further spreading of the infection.

Such households were enrolled in the study where at least one of the family members had recently been reported positive for SARS-CoV-2. All study participants provided written and informed consent before enrolment. For children under 18 years, written and informed consent was provided by the persons with care and custody of the children following aged-adapted participant information. The study was approved by the Ethics Committee of the Medical Faculty of the University of Bonn (approval number 085/20).

### 2.2. Sampling

Households were defined as people living together within one flat or one house with regular close contact with each other nearly every day. Twenty-one households with at least one person who recently tested positive for SARS-CoV-2 RNA were randomly selected and contacted by telephone. All persons who agreed to participate were visited at their homes. Throat swabs for virus diagnostics were obtained from all adults following previously published protocols [40]. Age, sex, and time of quarantine were recorded for all individuals living in each household. 

The main focus of this study was on sampling environmental media such as air, wastewater, and swab samples of different surfaces and fomites (consumer goods and furnishings) in accordance with the WHO “how-to” guide at that time as a reference [41]. Routine cleaning in every household was performed, on average, at least 48 h before sampling. Cleaning in households was carried out without the use of disinfectants. 

Air samples were obtained employing cyclone sampling [42] via Coriolis Micro—Air sampler (Bertin Technologies SAS, Montigny-le-Bretonneux, France). The air collectors were positioned in the middle of the room that was used most frequently by the residents, usually the living room or the kitchen (all the rooms had no ventilation equipment). According to Mallach et al. the position in the room should have no influence on the results of sampling [43]. During sampling, close contact with the air sampler (e.g., speaking within 2 m of the sampler) was avoided. Sample collection was performed with 300 L per minute for 10 min in 15 mL of 0.9% NaCl. Wastewater samples of 50 mL were obtained using sterile syringes and catheters to reach the wastewater in the siphons of sinks, showers, and toilets in bathrooms. Air and wastewater samples were stored and transported at +4 °C. 

Surface samples were taken using a swab with a synthetic tip and a plastic shaft (FLOQSwabs^TM^, Copan, Italy) and added 1 mL of 1× PBS (Gibco, Thermo Fisher Scientific, Schwerte, Germany) supplemented with 2% (*v*/*v*) FBS (Pan Biotech, Aidenbach, Germany). The research team defined the following surfaces and fomites of frequent and shared use to be tested: electronic devices (remote control, cell phone), knobs and handles, furniture and fixtures, and clothing. Furthermore, plants, animals, food, and drink were tested in specific cases of suspicion (i.e., close contact with pets, cut fruit left out for hours near symptomatic people). Dry surface swabs were performed from these items as described in Cheng et al. [44] and the swab tips stored at 4 °C for transport. At a maximum of 6 h after collection, swabs were resuspended as described above and all laboratory analyses were performed within 48 h.

### 2.3. Laboratory Analysis

All samples were transported to the virology laboratory within 6 h of sampling. Analysis was performed via qRT-PCR following previously published protocols [40]. Briefly, samples were homogenised by short vortexing and Viral RNA was extracted from 300 μL of the media containing samples on the chemagic™ Prime™ instrument platform (Perkin Elmer) using the chemagic Viral 300 assay according to manufacturer’s instructions. The RNA was used as template for three quantitative real-time RT-PCR reactions (SuperScript™III One-Step RT-PCR System with Platinum™ TaqDNA Polymerase, Thermo Fisher) to amplify sequences of the SARS-CoV-2 E gene24 (primers E_Sarbeco_F1 and R, and probe E_Sarbeco_P11), the RdRP gene (primers RdRP_SARSr_F, and R, and probe RdRP_SARSr-P21), and internal control for RNA extraction, reverse transcription, and amplification (innuDETECT Internal Control RNA Assay, Analytik Jena #845-ID-0007100). Samples were considered positive for SARS-CoV-2 if amplification occurred in both virus-specific reactions and if both the Ct of the internal control and an in vitro transcript (IVT) control of a defined concentration were in the expected range. The Ct of the IVT was referenced to a standard curve recorded earlier on the same device.

The isolation of infectious virus from environmental samples was attempted by seeding Vero E6 cells in 24-well plates or T25 flasks at a density of 70–80%. Cells were incubated with 200 µL (24-well)–1000 µL (T25 flask) of the sample material supplemented with 1× penicillin/streptomycin/amphotericin B and incubated for 1 h at 37 °C in 5% CO_2_. For wastewater samples, 10% (*v*/*v*) of inoculation volume was replaced by 10× PBS to obtain a final concentration of 1× PBS. Note that the wastewater samples were not concentrated before testing, neither before qRT-PCR nor before cell culture. After 1 h of incubation, the inoculum was removed and Dulbecco’s Modified Eagle’s medium (Gibco) with a 3% foetal bovine serum (Pan Biotech) and 1× penicillin/streptomycin/amphotericin B was added. Cells were incubated over several days at 37 °C and 5% CO_2_ and were observed for development of a cytopathic effect that typically occurs for growth of SARS-CoV-2 on Vero E6 cells. 

### 2.4. Statistical Analysis

Descriptive statistical analysis was performed via Stata IC 15.1 (StataCorp, College Station, TX, USA). For the household data, the total, the median, the interquartile range (IQR), and the range were reported. In total, 95%-confidence intervals (95%-CI) for proportions were calculated using the Wilson method [45].

The *χ*^2^-Test or Fisher exact test was used to analyse possible associations between subgroups of categorical variables. An α = 0.05 was considered statistically significant.

## 3. Results

### 3.1. Household Data

In total, data from 21 households were included in the analysis. Complete information was available for 58 study participants (43 adults and 15 children).

Table 1 shows the profile of all investigated households.

Pharyngeal swab samples tested positive for 26 out of 43 persons (60.47%) by qRT-PCR. The median number of adults testing positive was one per household (IQR: 1–2); in two households, no qRT-PCR-positive person was discovered. We obtained samples from 9 children, with 4 of them testing positive (44.44%). There was no association between adults and children who tested positive within our study group (exact test, *p* = 0.469).

### 3.2. Environmental Sampling Data

In total, 200 environmental samples (15 air samples (8%), 66 wastewater samples (33%), and 119 object swabs (59%)) from 21 households were included in the analysis. Table 2 shows the number of qRT-PCR-positive samples considering the sample type. Infectious virus could not be propagated in Vero E6 cells from any environmental sample.

Among environmental samples, as shown in Table 2, wastewater samples most commonly tested positive for SARS-CoV-2 RNA (15%). For further analysis, four wastewater subtypes were categorised as follows: washbasin siphons, shower siphons, toilets, and process water. Table 2 shows the positive samples within these subtypes. No significance between wastewater subtype and detection of SARS-CoV-2-status was observed (χ^2^-Test, *p* = 0.700).

In addition, the object samples were divided into the following six subtypes for further analysis: “electronic devices”, “knobs and handles”, “plants and animals”, “furniture and furnishings”, “foods and drinks”, and “clothing.” Table 2 shows the results of qRT-PCR analysis within the subtypes. There was no significant association between object subtype and qRT-PCR-status (χ^2^-Test, *p* = 0.843).

Four object samples were tested positive (3%), i.e., an electronic device (remote control), two metallic doorknobs, and one wooden chopping board (kitchen countertop).

No significant association between positive wastewater samples and positive object samples was observed (χ^2^-Test, *p* = 0.851).

### 3.3. Associations between Human and Environmental Data

Overall, no statistically significant association was found between the environmental samples that tested qRT-PCR positive and the transmission of the virus among household members (χ^2^-Test, *p* = 0.148). The households with positive environmental qRT-PCR results were further analysed with regard to the number of adults (χ^2^-Test, *p* = 0.249), the number of children (χ^2^-Test, *p* = 0.263), the proportion of females (χ^2^-Test, *p* = 0.410), the median age per household (χ^2^-Test, *p* = 0.453) and the time of quarantine (χ^2^-Test, *p* = 0.459). No correlation between qRT-PCR-positive environmental samples and qRT-PCR-positive human samples could be found in this study (χ^2^-Test, *p* = 0.756). There was no household with qRT-PCR-positive environmental samples and qRT-PCR-negative human samples. The number of positively tested members of all households is not associated with the number of positive samples from the households’ environment (exact test, *p* = 0.449).

## 4. Discussion

The role of environmental contamination as a potential way of SARS-CoV-2 transmission in quarantined households was unclear at the beginning of the SARS-CoV-2 pandemic in Germany. To that point, only data on contamination of the hospital patient environment existed, and it was assumed that transmission in households was negligible. In the high-prevalence community setting we investigated here, the persons who tested positive were sent to quarantine together with all their immediate family members as a precautionary measure, even if they were negative or had not been tested. This quarantine was maintained for 2 weeks without any testing.

All outbreaks of three or more cases occurred in an indoor environment, which confirms that sharing indoor space is a major SARS-CoV-2 infection risk [38]. Wu and colleagues stated that the only household risk factor is the person-to-person transmission between family members without protective measures, varying with the size of the household [46]. The infection rate of close contacts was 38% for households with 1 contact, 50% for households with 2 contacts, and 31% for households with 3 contacts [47]. Madewell and colleagues conducted a meta-analysis on studies of SARS-CoV2 transmission in the home [48]. The results of the meta-analysis were also confirmed by the findings of the additional study we conducted in the Heinsberg district [40]. Self-isolation from other household members of individuals testing positive was not queried in the present study. Therefore, the transmission may also occur due to close contact. According to Wu et al., several preventive in-household measures like separate dining, indoor isolation, ventilation and disinfection, index patient living alone, and wearing masks after index case symptom onset were not associated with COVID-19 prevalence [46]. Nevertheless, humans infected with SARS-CoV-2 can spread the contagious virus through respiratory droplets and aerosols. On the other hand, hard evidence regarding the indirect transmissibility of coronaviruses from contaminated surfaces and their persistence on surfaces are rare at present [49]. This study, therefore, aimed to determine the role of the domestic environment in quarantined households in SARS-CoV-2 transmission.

SARS-CoV-2, the causative agent of the COVID-19 pandemic, may be transmitted via airborne droplets or contact with surfaces onto which droplets have been deposited [50]. Several studies have already reported on the stability of SARS-CoV-2 on different surfaces under laboratory conditions or in hospitals as follows: Van Doremalen and coworkers detected viable viruses for up to 2–3 days on surfaces of plastic and stainless steel [7], and Chin and collaborators describe SARS-CoV-2 persistence of between two and seven days, depending on whether the surface is smooth. In contrast to this, we tested under field conditions in households with, at that time, state-of-the-art methods [51].

We were only able to detect viral RNA in 3% of all fomite samples and were not able to detect viral RNA in any air samples. In contrast, 15% of all wastewater samples were positive for SARS-CoV-2 RNA, which indicates that mouthwash in washbasins, body wash in the shower, and faeces in toilets and wastewater could pose a relevant risk for exposure. The low number of positive fomite and surface samples may also be due to methodological problems because the viral transport medium [43] might not be able to stabilise dried-up virus samples. Thus, the swab/transport solution combinations used might not have been suitable for keeping the formerly dry viral RNA stable until it was analysed in the laboratory, as opposed to a virus in watery or mucus solution. Assuming that the results are not methodologically inappropriate, they could indicate that the survival of SARS-CoV-2 in the domestic environment may not be as long as under the laboratory conditions described by Chin and coworkers (<2 days on wood and clothing, <4 days on smooth surfaces, <7 days on steel or plastic) [51]. Additionally, in terms of transmission via surfaces, it is important to consider that the circumstances of natural infection, i.e., direct delivery of a smear sample to the human mucosa, are vastly different from cell culture conditions and not necessarily more detrimental to transmission. Likewise, the propagatability of the virus from a sample in vitro would have only shown potential infectiousness, albeit as close to a proof as possible barring animal experiments.

Following international recommendations, air samples should be taken indirectly as swabs of air purifier ventilation outlets [41]. Since this is just a surrogate for real air contamination and, normally, households in Germany are not equipped with ventilators or air purifiers, cyclone air samplers were used. Cyclone samplers may be less efficient than other sampler types at recovering low concentrations of airborne viruses due to the physical stress caused by centrifugal force [52]. Yet, several studies using cyclone air collectors of the same type to investigate air contamination in isolation rooms of hospitals showed that the air sampler was able to detect SARS-CoV-2 RNA in up to 38.7% of the samples [44,53,54]. However, in contrast to the present study, these investigations were conducted in hospitals or homes for long-term care, where a higher viral load is to be expected. In contrast, Ong and colleagues found all air samples taken on a ward with COVID-19 patients were negative despite proximity to infected patients [16]. Further experimental investigations of different air samplers in defined environments and preferably including households of the general population households would be necessary to exclude a method-related false low recovery rate. A review by Borges and colleagues addresses this issue [55].

SARS-CoV-2 can be sustained in the air in closed, unventilated rooms (e.g., bathrooms without windows) for at least 30 min without losing infectivity [49]. Furthermore, room humidity impacts the survival time of viruses in general. Accordingly, droplets or aerosols with SARS-CoV-2 in a viable and infectious form can be formed, while flushing open toilets without closed lids or arising from contaminated siphons and thus could represent a possible transmission pathway. At the moment, only 2–10 percent of confirmed SARS-CoV-2 cases have been associated with diarrhoea [29,30,31]. All published information on the faecal spread of SARS-CoV-2 derived from hospitalised patients [25,27,56,57,58], and rarely with data of mild cases [59,60]. Wang and coworkers reported the detection of viral RNA in respiratory tract swabs and stools with 44/153 positive faecal samples. Four positive faecal samples showed high copy numbers with a mean cycle threshold (Ct) value of 31 4 (<2.6 × 104 copies/mL) [26].

Probably due to the rather high cycle threshold (CT) values > 30 obtained in the qRT-PCR analysis of the samples in this study (Supplementary Figure S1), virus isolation in cell culture has not been successful so far in our laboratory. This could indicate that transmission via those surfaces tested here is unlikely. Furthermore, several wastewater samples had a toxic effect on the Vero E6 cells used for propagation, which may be linked to detergent residues. Thus, it is difficult to give specific hygienic behaviour precautions but rather recommend basic hygiene measures for dissemination prevention [61]. Only a few other studies reported the presence of viral RNA in the faeces of patients with the virus that remained propagatable for several hours in faeces and 3–4 days in urine, respectively [37,56]. The active virus was also found in the faeces of patients without diarrhoea, suggesting a systemic infection [26]. Viral loads showed that shedding from the gastrointestinal tract may be higher and longer-lasting compared to the respiratory tract. Yet, studies report that SARS-CoV-2 from cultured stool samples is rarely found [39]. Thus, the risks associated with faecal transmission of SARS-CoV-2 and whether this respiratory virus can be disseminated by enteric transmission still need to be determined [62].

Concerning food, faeces, and wastewater, which are highly heterogeneous based on their basic properties, sample collection and processing is particularly difficult to standardise [63]. With regard to the results of the wastewater samples in our study, the percentage of SARS-CoV-2 RNA positive samples was lowest in toilets (8.70%), higher in shower siphons (18.75%), and highest in washbasin siphons (19.23%). The presence of the viral RNA in the wastewater, even at low levels, indicates that SARS-CoV-2 can remain detectable in sewage for days or weeks [64,65,66]. Thus, the wastewater system is serving as a surveillance system for the circulation of the virus within several environments [35,67,68].

In general, wastewater requires special hygienic attention, for example, with regard to multidrug-resistant bacteria and antibiotic residues [69,70,71,72], enteric viruses like norovirus or rotavirus [73], and coronavirus [74]. The enteric transmission of SARS-CoV led to a large outbreak cluster in Hong Kong in 2003 [75]. Aerosols containing SARS-CoV were transferred in the building complex of Amoy Gardens through the plumbing system and entered the bathrooms of uninfected individuals via floor drains. The index case of the outbreak had a high viral load in faeces and urine that might have become aerosolised. Of all the reported cases of SARS-CoV in Hong Kong in 2003, the Amoy Gardens outbreak accounted for 18% [76]. Although SARS-CoV-2 has not yet been cultured from sewage, enteric dissemination of and exposure to SARS-CoV-2 via sewage is also considered a notable risk [62]. Therefore, existing hygiene recommendations (washing hands after contact with wastewater, flushing the toilet with the lid closed, and avoiding re-contamination of drinking water systems and the domestic environment with wastewater) are considered to be necessary to sufficiently control this transmission route. Furthermore, preventive and intervention measures should not start at the wastewater treatment in the treatment plant but already in the immediate surroundings of the patient, to minimise the infection potential.

## 5. Conclusions

While SARS-CoV2 is predominantly transmitted by air, the domestic environment might play a role in transmission only in the case of high viral loads. Should follow-up studies find higher RNA concentrations on domestic surfaces, their in vitro propagatability should definitely be tested for. Even if surfaces and fomites did not show a high contamination rate in this study, the detection of viral RNA in the wastewater of washbasins, showers, and toilets showed significantly higher contamination with SARS-CoV-2.

## Figures and Tables

**Table 1:** Household data.

	Total	Per Household		
		Median	IQR	Range
Number of households	21			
Number of adults (≥18)	43	2	2–2	1–4
Number of children (<18)	15	0	0–2	0–3
Proportion of females (%)	51.72	50.00	50.00–66.67	0.00–100.00
Median household age (years)		31.00	28.00–53.00	9.50–75.00
Time of quarantine (days)		5	5–6	0–6

**Table 2:** Overview of qRT-PCR-positive sample types.

Sample Type		n	qRT-PCR-Positive		
			n	%	(95%-CI)
Air samples		15	0	0%	
Wastewater samples		66	10	15%	(8; 26)
	Washbasin siphons	26	5	19%	(9; 38)
	Shower siphons	16	3	19%	(7; 43)
	Toilet	23	2	9%	(2; 27)
	Other	1	0	0%	
Object samples		119	4	3%	(1; 8)
	Electronic devices	52	1	2%	(0; 10)
	Knobs and handles	31	2	6%	(2; 21)
	Plants and animals	11	0	0%	
	Furniture and fixtures	19	1	5%	(1; 25)
	Foods and drinks	4	0	0%	
	Clothing	2	0	0%	
Total		200	14	7%	(4; 11)

## Data Availability

All data will be made available upon reasonable requestle request.

## Supplementary Materials

The following are available online at https://www.mdpi.com/article/10.3390/v14051075/s1, Figure S1: Ct values of environmental samples collected in households.
