# Supplementary material for: SARS-CoV-2 in Environmental Samples of Quarantined Households
Source: Viruses. 2022 May 17;14(5):1075. doi: 10.3390/v14051075 (PMC9147922; doi:10.3390/v14051075)
Supplement: Supplementary file 1 [file viruses-14-01075-s001.zip › viruses-1680397-supplementary.pdf]

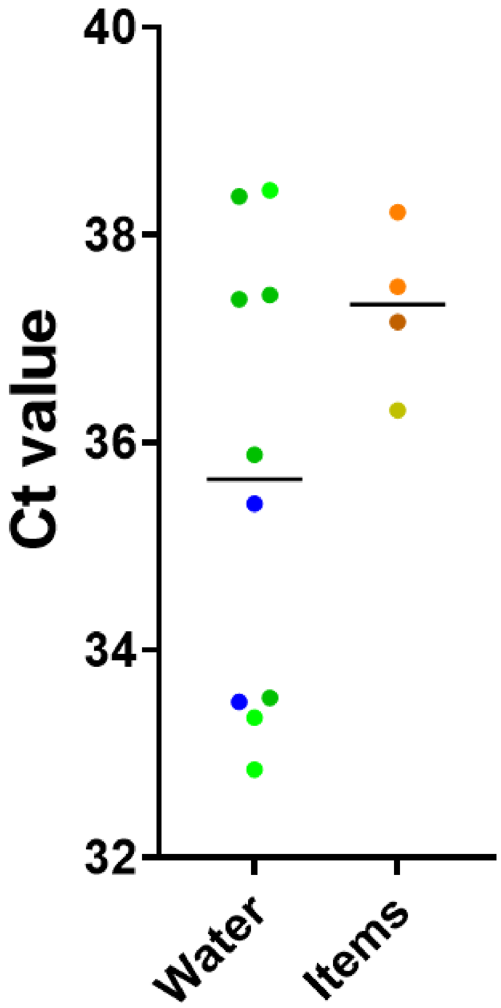

Figure S1: Ct values of environmental samples collected in households. The E-gene Ct values of those samples that were positive in SARS-CoV-2 RT-PCR from all samples listed in Table 2 are shown, subdivided by the nature of the samples. Water samples include washbasin (dark green), shower (green), and toilet water (blue), item samples include swabs of a remote control (yellow), 2 door handles (orange), and a stairway railing (brown).
